# Unveiling Novel Urease Inhibitors for *Helicobacter pylori*: A Multi-Methodological Approach from Virtual Screening and ADME to Molecular Dynamics Simulations

**DOI:** 10.3390/ijms25041968

**Published:** 2024-02-06

**Authors:** Paulina Valenzuela-Hormazabal, Romina V. Sepúlveda, Melissa Alegría-Arcos, Elizabeth Valdés-Muñoz, Víctor Rojas-Pérez, Ileana González-Bonet, Reynier Suardíaz, Christian Galarza, Natalia Morales, Verónica Leddermann, Ricardo I. Castro, Bruna Benso, Gabriela Urra, Erix W. Hernández-Rodríguez, Daniel Bustos

**Affiliations:** 1 Departamento de Farmacología, Facultad de Ciencias Biológicas, Universidad de Concepción, Concepción 4030000, Chile; paulinvalenzuela@udec.cl; 2 Center for Bioinformatics and Integrative Biology, Facultad de Ciencias de la Vida, Universidad Andres Bello, Av. República 330, Santiago 8370146, Chile; romina.sepulveda@unab.cl; 3 Núcleo de Investigación en Data Science, Facultad de Ingeniería y Negocios, Universidad de las Américas, Santiago 7500000, Chile; malegriaa@udla.cl; 4 Doctorado en Biotecnología Traslacional, Facultad de Ciencias Agrarias y Forestales, Universidad Católica del Maule, Talca 3480094, Chile; elizabeth.valdes@alu.ucm.cl (E.V.-M.); victor.rojas.03@alu.ucm.cl (V.R.-P.); 5 Biomedical Research Labs, Facultad de Medicina, Universidad Católica del Maule, Talca 3480094, Chile; ileanag@ucm.cl; 6 Departamento de Química Física, Facultad de Ciencias Químicas, Universidad Complutense de Madrid, 28040 Madrid, Spain; reysuard@ucm.es; 7 Departamento de Matemáticas, Facultad de Ciencias Naturales y Matemáticas, Escuela Superior Politécnica del Litoral, Guayaquil 090112, Ecuador; chedgala@espol.edu.ec; 8 Magíster en Ciencias de la Computación, Universidad Católica del Maule, Talca 3460000, Chile; nmoralesr@ucm.cl (N.M.); vlleddermann@ucm.cl (V.L.); 9 Multidisciplinary Agroindustry Research Laboratory, Instituto de Ciencias Aplicadas, Facultad de Arquitectura, Construcción y Medio Ambiente, Universidad Autónoma de Chile, Cinco Pte. N°1670, Talca 3467987, Chile; ricardo.castro@uautonoma.cl; 10 School of Dentistry, Faculty of Medicine, Pontificia Universidad Católica de Chile, Santiago 7810000, Chile; bruna.benso@uc.cl; 11 Laboratorio de Bioinformática y Química Computacional, Departamento de Medicina Traslacional, Facultad de Medicina, Universidad Católica del Maule, Talca 3480094, Chile; gabriela.urra@alu.ucm.cl; 12 Unidad de Bioinformática Clínica, Centro Oncológico, Facultad de Medicina, Universidad Católica del Maule, Talca 3480094, Chile

**Keywords:** computer-aided drug design, structure-based virtual screening, pharmacophore-based virtual screening, molecular dynamics simulations, ADMET, urease, *Helicobacter pylori*

## Abstract

*Helicobacter pylori* (*Hp*) infections pose a global health challenge demanding innovative therapeutic strategies by which to eradicate them. Urease, a key *Hp* virulence factor hydrolyzes urea, facilitating bacterial survival in the acidic gastric environment. In this study, a multi-methodological approach combining pharmacophore- and structure-based virtual screening, molecular dynamics simulations, and MM-GBSA calculations was employed to identify novel inhibitors for *Hp* urease (*Hp*U). A refined dataset of 8,271,505 small molecules from the ZINC15 database underwent pharmacokinetic and physicochemical filtering, resulting in 16% of compounds for pharmacophore-based virtual screening. Molecular docking simulations were performed in successive stages, utilizing HTVS, SP, and XP algorithms. Subsequent energetic re-scoring with MM-GBSA identified promising candidates interacting with distinct urease variants. Lys219, a residue critical for urea catalysis at the urease binding site, can manifest in two forms, neutral (LYN) or carbamylated (KCX). Notably, the evaluated molecules demonstrated different interaction and energetic patterns in both protein variants. Further evaluation through ADMET predictions highlighted compounds with favorable pharmacological profiles, leading to the identification of 15 candidates. Molecular dynamics simulations revealed comparable structural stability to the control DJM, with candidates 5, 8 and 12 (CA5, CA8, and CA12, respectively) exhibiting the lowest binding free energies. These inhibitors suggest a chelating capacity that is crucial for urease inhibition. The analysis underscores the potential of CA5, CA8, and CA12 as novel *Hp*U inhibitors. Finally, we compare our candidates with the chemical space of urease inhibitors finding physicochemical similarities with potent agents such as thiourea.

## 1. Introduction

*Hp* is a Gram-negative bacterium with a helical structure that allows motility. This microorganism is also able to change from helical to coccoid to survival in the gastric microenvironment of its host [1]. Additionally, it has the ability to form biofilms in order to decrease susceptibility to antibiotics, resulting in mutations in its genome related to antibiotic resistance and difficulties in eradicating the bacteria [2]. Over 50% of the global population is estimated to be infected with *Hp*, with notable variations in prevalence among countries and within different regions of the same country. Africa has the highest *Hp* infection rate, with a prevalence of 87% in South Africa and 91% in Nigeria [3,4]. *Hp* treatment usually involves antibiotic-containing triple therapy with clarithromycin, amoxicillin, and metronidazole. However, these bacteria can be resistant to clarithromycin. Second line treatments include triple therapy with levofloxacin and quadruple therapy with bismuth [5]. In recent times, awareness has begun to increase regarding antibiotic resistance, as no available therapy can eradicate *Hp* infections in all patients. Therefore, emphasis should be placed on discovering new drugs and therapeutic approaches [6]. *Hp* secretes a large amount of urease, an enzyme that catalyzes the hydrolysis of urea into carbon dioxide and ammonia which neutralize the acidic gastric pH, altering the gastric mucosa layer and allowing the bacterium to survive in the stomach [7]. The urease of *Hp* (*Hp*U) is a nickel-dependent metalloenzyme with a dodecameric structure containing two subunits [8,9]. *Hp*U is considered an important virulence factor involved in aiding and triggering the colonization of the gastric epithelium and an inflammatory immune response, among various processes [10].

Nitrogen-based fertilizers are crucial for agriculture as they are one of the main nutrients required for plant growth and development. Urea is one of the most widely used nitrogen fertilizers worldwide, with a nitrogen mass ratio of 46%, and can be found at low cost [11]. The application of urea as a fertilizer also brings certain environmental concerns due to the formation of pollutants, eutrophication and soil acidification, and water contamination resulting from the hydrolysis of urea by ureases present in the soil microbiota [12]. Inhibiting the catalytic action of urease is a promising target for therapy in humans and animals, as well for pesticide design. Multidisciplinary approaches are needed to analyze the enzyme’s structure, ligands, and activity regulators and might form a blueprint for the development of inhibitors [13,14].

Several studies have been undertaken over the last decade to discover possible therapeutics for urease-associated problems by examining urease enzyme inhibition. Coumarins are the most basic members of the oxygen heterocycle category (benzo-α-pyrones). These compounds are known for their unique chemical structure and low toxicity. The major coumarin groups formed by simple coumarins rings, furocoumarins, pyranocoumarins, n-methylcoumarins, and biscoumarins have been demonstrated to exhibit urease inhibitory activity [15].

**Figure 1:**
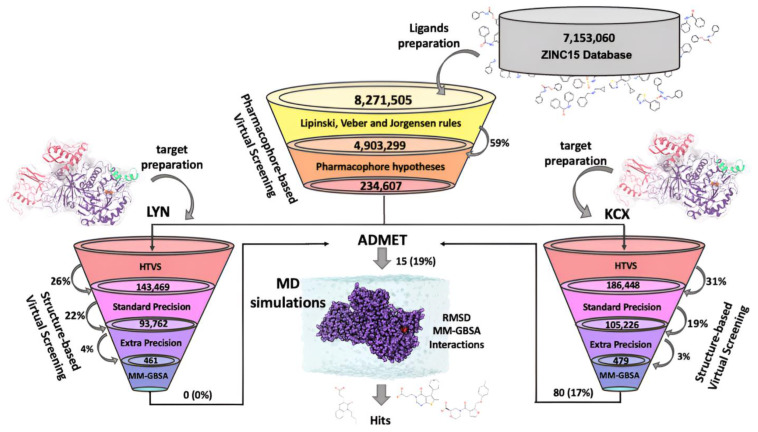
Schematic workflow: Each stage is represented as a section of a funnel receiving as input the data processed by the output of the previous stage. The number of molecules processed in each stage is shown, as well as the percentage with respect to the total of the immediately previous stage.

In this theoretical work, we combine pharmacophore- and structure-based virtual screening (PBVS and SBVS), molecular dynamics simulations (MDs), end-point molecular mechanics-generalized born surface area (MM-GBSA) calculations and administration, distribution, metabolism, and excretion (ADME) filters and apply them to 7 million compounds from the ZINC15 database [16] to discover new candidate inhibitors for *Hp*U (See protocol in Figure 1).

## 2. Results and Discussion

### 2.1. Database Curation

The ZINC15 database was obtained in mol2 format by selecting molecules that were available for sale and in stock. The download was limited to compounds with a partition coefficient (LogP) of 5 or less and a molecular weight (MW) of 500 g/mol or less, resulting in a total of 7,153,060 molecules. Upon conducting an exploratory analysis of the downloaded files, it was observed that a significant number of molecules with identical identifiers or names existed within the ZINC15 database. These molecules were found to be tautomers, constituting approximately 7.5% of the downloaded database. These duplicate molecules were removed to streamline the dataset, retaining only one representative from each group of repeating molecules. As a result, the library of compounds was reduced to 6,619,086 molecules. Subsequently, the prepared molecules underwent processing using the LigPrep tool, which is part of the Maestro–Schrödinger suite [17]. A maximum of 2 poses were generated for each molecule, leading to a final count of 8,271,505 molecular configurations.

The set of molecules prepared with LigPrep underwent a series of filtering steps based on pharmacokinetic and physicochemical criteria. These filters are designed to adhere to the “Rule of Five”. By applying these filters, the database is reduced by 20%, resulting in 6,591,236 molecules, allowing at most only one violation of any of the four rule parameters. Concerning the MW, only molecules with less than 500 g/mol were selected. Regarding the LogP, molecules with a value equal to or less than 5 were included. As for hydrogen bond (HB) acceptors, molecules with a maximum of 10 acceptors were selected.

Finally, for HB donors, only molecules with a count ranging from 0 to 5 were retained (Figure S1 in Supporting Information material). Furthermore, the dataset underwent an additional filtration step based on Jorgensen’s “Rule of 3”. This rule further refined the database, reducing it to 5,018,768 molecules. This represents a 24% decrease compared with the previous filtering step. Any molecule violating one or more of the rule’s criteria were excluded in this case. Regarding aqueous solubility (LogS), molecules with a cutoff value greater than −5.7 were considered. Similarly, only molecules with a cutoff greater than 22 nm/s of permeability in Caco-2 cells were retained. Lastly, concerning the number of primary metabolites, molecules with a count ranging from 0 to 7 were considered (Figure S2). As a third criterion, the “Veber Rule” was applied, ensuring that no violations occurred. This filtering step resulted in a total of 4,903,299 molecules, representing a 2% reduction compared with the previous rule. Specifically, the number of rotatable bonds (RotB) was considered, with a cutoff value of 10 or less. In terms of polar surface area (PSA), molecules with a PSA value equal to or less than 140 Å^2^ were retained. Lastly, the sum of HB acceptors and HB donors was limited to 12 or less (Figure S3). Applying the three aforementioned rules resulted in a final count of 4,903,299 molecules for this particular virtual screening (VS) layer. These molecules constitute approximately 59% of the initial database containing prepared molecules.

### 2.2. Pharmacophore Hypothesis

**Figure 2:**
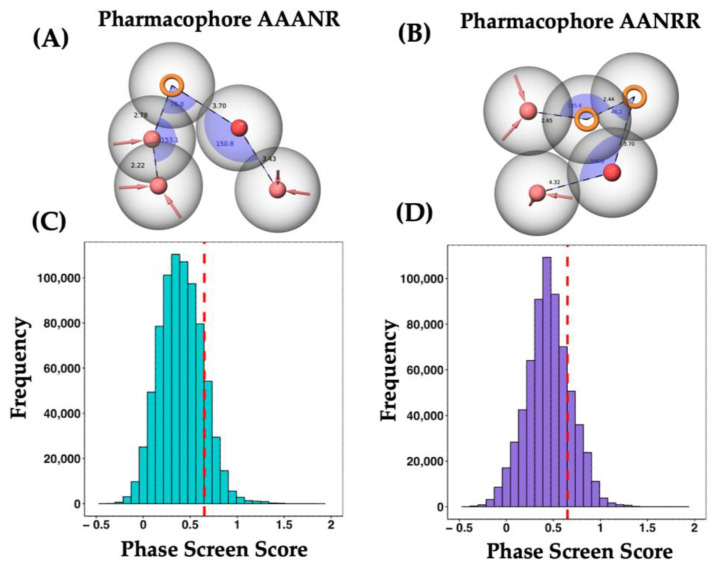
Pharmacophore-based virtual screening: The best two pharmacophore hypotheses (**A**,**B**). The pink and red spheres represent HB donors and negatively charged groups, while the rings correspond to aromatic groups. The alignment of each molecule in the database (**C**,**D**) is shown against their respective pharmacophore hypotheses (**A**,**B**). The similarity measured is represented by the term PSS, the more positive is the score, the more similar. The dotted red lines represent the cutoff value used to differentiate the selected molecules from those discarded.

The PBVS protocol, in conjunction with the phase tool [18] from the Maestro–Schrödinger suite, was utilized to generate 10 hypothesized pharmacophores using a set of coumarin with ureolytic activity as templates. The best two pharmacophore hypotheses were chosen based on the fulfillment of the following two criteria: (1) the best possible BEDROC score and (2) the highest number of physicochemical criteria, selecting an AANRR score of 0.985 and an AAANR score of 0.980 (where A is an HB acceptor, N is a negative group, and R is an aromatic ring) (Figure 2A,B). The BEBROC score is a metric that factors in the number of characteristics (with a preference for those with more features), and the absence of significant similarity in distances, angles, and characteristics between the hypotheses. The BEDROC score, a statistical value used in VS, falls within the range of [0, 1] and represents the probability of classifying an active compound more efficiently than a randomly selected compound. This approach facilitates the discrimination of molecules that conform to those specific characteristics. Lastly, the spatial geometry of the chosen hypotheses was examined to ensure that the two selected pharmacophores did not closely align in terms of distances and angles. In the process of generating pharmacophore hypotheses, emphasis was given to molecules featuring coumarin nuclei capitalizing on the chemical diversity offered by the presence of 142 different substituent groups, including coumarins and bis-coumarins. Subsequently, both pharmacophore hypotheses were applied to a compound library comprising 4,903,299 molecules. Among these, 1,429,460 molecules displayed similarity to the employed pharmacophores, accounting for approximately 29% of the total aligned molecules. When considering each hypothesis individually, the AAANR and AANRR hypotheses yielded 772,351 and 657,109 molecules, respectively. The “Phase Screen Score” (PSS) term represents the degree of similarity between the compound and the pharmacophore hypothesis. Here, PSS ranges from −0.45 to 2.00, with higher values indicating a greater resemblance to the pharmacophore. A cutoff value is then applied to select molecules based on their PSS. The distribution of molecules in the compound library can be observed for each set. For the AAANR pharmacophore set, there are 107,930 molecules with a PSS above the threshold value. Similarly, for the AANRR set, 126,677 molecules surpass the threshold (Figure 2C,D). Considering both sets, the total number of molecules in the new library amounts to 234,607. This represents approximately 16% of the compounds evaluated in this stage.

### 2.3. Structure-Based Virtual Screening

In order to evaluate the ability of the glide program [19,20] to reproduce the crystallographic poses, we used the following three classic *Hp*U inhibitors: DJM (2-{[1-(3,5-dimethylphenyl)-1H-imidazole-2-yl]sulfanyl}-N-hydroxycetamide), BME (beta-mercaptoethanol) and HAE (ace-tohydroxamic acid) on the following, respective PDB structures ids: 6ZJA, 6QSU [21], and 1E9Y [22]. The process consists of eliminating the co-crystallized inhibitor from the urease binding site and docking it again (redocking), making a structural comparison using root mean square deviation (RMSD) and the binding energy obtained if the program manages to reproduce the experimental state. Table S1 shows that glide is able to reproduce the crystallographic pose in an acceptable way with an RMSD value below 3.0 Å for the best crystallographic poses based on docking energy.

Subsequently, the strategy employed for the identification of potential candidate molecules targeting both urease variants (LYN and KCN) from a database of 234,607 compounds involved a multi-stage approach employing molecular docking simulations. These simulations were carried out in successive rounds, progressively increasing the exhaustiveness parameters, including HTVS, SP, and XP of the glide program [19,20] as is depicted in Figure S4. During the initial high-throughput virtual screening (HTVS) stage, 552,716 poses were generated for the LYN variant, and a total of 600,588 poses were obtained for the KCX variant (refer to Table 1). In both cases, a cutoff based on the first quartile (−4.5 kcal/mol) was applied to select the most promising candidates, resulting in 156,814 and 196,349 molecules for LYN and KCX, respectively. Subsequently, molecules with predicted positions outside the binding site were eliminated, leading to a refined set of 143,469 for LYN (26% of the total poses) and 186,448 for KCX (31% of the total poses). The subsequent round of docking simulations using a standard precision (SP) algorithm yielded 429,972 and 557,590 poses for LYN and KCX, respectively. Applying a cutoff of −6.5 kcal/mol enabled the retention of 93,829 and 105,364 molecules for LYN and KCX, respectively. Further refinement, involving the exclusion of molecules located outside the catalytic site, resulted in final candidate sets of 93,762 (22% of the total) for LYN and 105,226 (19% of the total) for KCX. The final docking stage employing an extra precision (XP) algorithm produced 267,641 and 297,494 docking poses for LYN and KCX, respectively. Using a cutoff of −8.5 kcal/mol, the candidate pools were reduced to 11,368 (4%) for LYN and 16,160 (5%) for KCX. Importantly, no poses were found outside the binding site at this stage.

Figure S4 illustrates the energy distribution for each docking stage in LYN and KCX variants. These distributions highlight the superior median value of the XP method compared with the HTVS and SP algorithms, underscoring the enhanced discriminatory power of XP in minimizing false positives and establishing a robust correlation between favorable poses and high scores. In essence, the XP algorithm was designed to streamline the identification process, ensuring a more accurate selection of potential drug candidates [20].

Three additional refinement filters were implemented. Firstly, molecules or poses with duplications were eliminated, retaining only the most energetically favorable instance, resulting in a reduction to 1337 and 1609 molecules for LYN and KCX, respectively. Secondly, molecules positioned more than 3.5 Å away from any of the nickel ions were excluded, further refining the selection to 1188 for LYN and 1471 for KCX. Thirdly, a comparative analysis using RMDS was employed, retaining only those molecules with a value greater than 0, thereby eliminating tautomers and preserving only the most energetically favorable structures.

Upon applying these filters, the final count of molecules in this protocol phase amounted to 461 and 479 for LYN and KCX, respectively, comprising 4% and 3% of the total candidates. This stringent filtration process ensures a highly selective set of potential drug candidates, emphasizing both energetic favorability and structural proximity to the target ions, while eliminating duplicates and tautomeric forms. The resulting subset represents a refined pool of molecules poised for further in-depth analysis.

It is essential to highlight the existence of a subset of molecules derived from the preceding step, which is present in both set of molecules interacting with LYN and KCX variants. We denote this subset as “Both_KCX” for molecules forming a complex with the KCX protein and “LYN_Both” for the same molecules but interacting with the LYN protein. Notably these molecules may exhibit distinct interaction patterns in each variant. Additionally, we have used the nomenclatures “LYN_Only” and “KCX_Only” for molecules exclusively interacting with one of the enzyme variants.

To further refine the selection, an energetic re-scoring was conducted using the MM-GBSA technique. This method involved flexible treatment of all atoms within the protein structure located within a 5 Å radius from the ligand. MM-GBSA calculations were performed on the four distinct groups of complexes mentioned earlier (LYN_Only, LYN_Both, KCX_Only, KCX_Both). This comprehensive approach allows for a nuanced assessment of the energetics associated with ligand binding in the context of different protein variants, aiding in identifying high-affinity and selective compounds within each subset. Additionally, we calculate the interaction energies of the classic urease inhibitors (UIs)—DJM, HAE, BME and the natural substrate urea—as controls.

The Cheng–Prusoff is an equation employed in pharmacology to calculate the inhibition constant (*Ki*) of an enzyme inhibitor, based on the concentration of inhibitor required to produce a specific effect and the substrate affinity constant (Km) of the enzyme. This equation proves particularly valuable in studies of competitive inhibition, as is the case for these inhibitors. The Cheng–Prusoff equation is sometimes empirically related to the affinity energy (ΔG). This relationship is based on the Cheng–Prusoff inhibition equation and the Gibbs–Helmholtz equation.

The equation expressing the relationship between the inhibition constant (*Ki*) and the affinity energy (ΔG) is as follows:

(1) $$\Delta G=RT\;ln\;K_{i}$$

The same approximation can be made for IC*_50_* values, replacing $K_{i}$ for IC_50_. The IC_50_ values reported for DJM and BME [21] and HAE [23] were transformed to the experimental ΔG ($\Delta G_{Experimental}$) by using Equation (1) and were compared with the binding affinities obtained theoretically for the KCX and LYN variants (Table S2). The MM-GBSA method, while valuable for predicting relative free energies to rank compounds, presents several notable limitations. One significant shortcoming is its omission of the entropic component in the calculation of Gibbs free energy. The method lacks the capability to accurately predict absolute free energies, focusing instead on rank compounds based on the relative energies between different compounds. This limitation diminishes its suitability for providing precise estimations. As a consequence, our predictions of affinity energy, while falling short in the precise prediction of the intricacies of absolute binding free energy, successfully ascertain the relative ranking of inhibitors employed as controls, i.e., DJM > HAE > BME (left to right in order of potency).

Notably, DJM surpasses the binding energy of the substrate urea by a significant margin, exhibiting a difference of −42.8 kcal/mol for LYN and −24.7 kcal/mol for KCX. It is important to highlight that the protein conformation used in these evaluations corresponds specifically to the structure solved in the complex with DJM.

**Figure 3:**
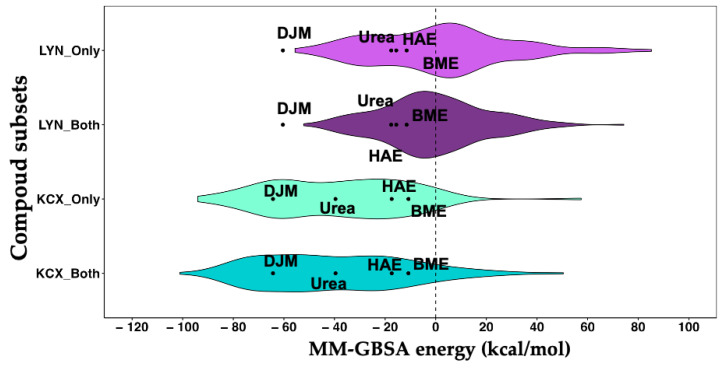
Binding free energy distribution of the compound subset: The distributions of free energy for the LYN_Only and LYN_Both subsets are visually represented by light purple and dark purple hues, respectively. Similarly, the free energy distributions for the KCX_Only and KCX_Both subsets are depicted using light and dark cyan colors, respectively. The free energy values for the control compounds (DJM, HAE, BME, and urea) are depicted as individual points within each violin plot. The inclusion of a dashed line at 0 kcal/mol serves to delineate differences in the proportions of complexes within each subset that exhibit unfavorable and favorable energy values.

Examining the free energy distributions within each subset (Figure 3), particularly for both LYN variants (LYN_Both and LYN_Only), it becomes apparent that a significantly larger proportion of protein–ligand complexes exhibit unfavorable values (above 0 kcal/mol) compared with the subset associated with the KCX variant. Specifically, the LYN_Both subset has 113 complexes with energies greater than 0 kcal/mol, while the LYN_Only subset 98 such complexes. In contrast, the KCX variant subset shows a notably lower count, with only 15 complexes in KCX_Both and 11 in KCX_Only having unfavorable values (Table S3). This observation underscores a distinct thermodynamic profile between the LYN and KCX variants, spotlighting a higher occurrence of energetically unfavorable interactions in the LYN subsets than their KCX counterparts.

Furthermore, by employing the free energy of the most favorable control, DJM, as a cutoff value (−60.4 kcal/mol in the LYN variant, Table S2), it becomes apparent that none of the evaluated compounds surpass the inhibitory potency exhibited by DJM in the LYN subsets. Concerning the subsets associated with KCX, it is noteworthy that KCX_Both exhibits 38 complexes with a more favorable affinity energy compared with the DJM control. Similarly, Table S3 shows that, within the KCX_Only subset, there are 36 complexes demonstrating better affinity than the DJM control, and 38 complexes for the KCX_Both subset with affinity energies superior to the control (DJM). These sets of 74 molecules then advance to the next stage of the drug discovery protocol for urease.

### 2.4. ADME Predictions

The previously mentioned subsets, KCX_Both and KCX_Only, underwent a comprehensive evaluation through a set of predictors for pharmacokinetic and pharmacodynamic characteristics, including Lipinski, Jorgensen, Veber, Ghose, Egan, and Muegge criteria. Additionally, the analysis incorporated assessments for structural alerts such as pan assay interference compounds (PAINS) and lead-likeness. By integrating these predictive tools, the aim is to prioritize compounds that show promising binding affinity and demonstrate the potential for effective drug development by meeting key criteria for bioavailability, metabolism, and safety. This multi-faceted approach ensures a more holistic assessment of the candidate compounds, contributing to the selection of molecules with optimal pharmacological profiles for further development within the drug discovery process used for the discovery of newer, more powerful, and safer UIs. The total number of violations across all evaluated parameters for each molecule was calculated. Remarkably, seven compounds within the KCX_Both subset (refer to Table S4) and eight compounds within the KCX_Only subset (refer to Table S5) exhibited no violations of any evaluated rule. These outstanding compounds were designated as CA# (candidate number) for the final stage of this protocol.

### 2.5. Molecular Dynamics Simulations

These final candidates (CA1 to CA15), along with the protein and nickel ions, underwent a 100 ns simulation in an NPT ensemble. Additionally, simulations were conducted for the urease–DJM, urease–HAE and urease–BME complexes [21]. A visual inspection of all trajectories confirmed that none of the compounds tested left the urease binding site at the simulated time. The RMSD profiles (Figure S5) across the entire simulation trajectory demonstrate that the DJM control is more thermodynamically stable than HAE and BME. These controls, DJM, HAE and BME, have respective median RMSD values close to 2.33 Å, 2.56 Å and 4.73 Å and respective interquartile ranges (IQR) of 0.43 Å, 0.45 Å and 0.87 Å. Notably, several candidate compounds exhibit similar median and IQR values, indicating their comparable structural stability during the simulations, as follows:

CA1: Median RMSD of 2.76 Å and IQR of 0.24 Å.
CA5: Median RMSD of 2.31 Å and IQR of 0.62 Å.
CA6: Median RMSD of 1.97 Å and IQR of 0.66 Å.
CA8: Median RMSD of 2.18 Å and IQR of 0.24 Å.
CA12: Median RMSD of 2.30 Å and IQR of 0.26 Å.

It is noteworthy that, while CA10 has a similar median RMSD to DJM (2.34 Å), it exhibits a considerably higher IQR (0.67 Å).

In a similar manner to that shown in the comparison of $\Delta G_{Experimental}$ and the values obtained with docking and MM-GBSA (Table S2), the combination of MDs and MM-GBSA allows the three controls to be correctly ranked when the median, computed over the entire trajectory of MDs, is considered (Table S6). Our data suggest the importance of considering the energetic evaluation of different conformational states of the complexes studied. This is in contrast with metrics such as top-scoring, in which a cherry picking is performed with the most favorable energy per molecule. When we consider the top-scoring (minimum value in Table S6), the computational prediction is not able to correctly rank the relative affinity of the HAE and BME compounds, overestimating the latter as a better inhibitor, when in practice it is not. These analyses corroborate the robustness of combining MDs and MM-GBSA, allowing an evaluation of the dynamic changes of the different conformations adopted by the *Hp*U binding site in the presence of the inhibitor and thus achieving a more accurate prediction when the complete ensemble is considered. Our data show that DJM is almost 38 kcal/mol and 46 kcal/mol more favorable than HAE and BME, respectively.

**Figure 4:**
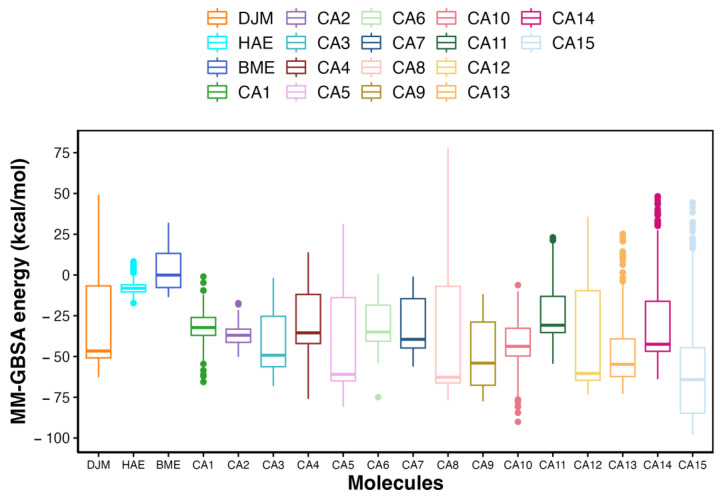
Binding free energy of the urease inhibitor candidates: Each boxplot in the analysis was constructed using the free energy values of the protein–ligand complex over 200 frames, corresponding to the 100 ns simulation period. The binding free energy of the inhibitor DJM, HAE and BME is presented as control.

The assessment of free energy values (Figure 4) for the protein–ligand complexes throughout the entire simulation period reveals distinctive behavior among the candidate compounds compared with the controls. Each candidate has a median $\Delta G_{Theoretical}$ more favorable than HAE and BME. However, DJM has a median $\Delta G_{Theoretical}$ of −46.6 kcal/mol and a IQR of 44.2 kcal/mol. Considering both median values and IQR provides a comprehensive understanding of the central tendency and variability in the free energy profiles of these candidate compounds throughout the simulation, aiding in the evaluation of their potential as UIs.

CA5:

- Median free energy almost 14.4 kcal/mol more favorable than DJM (−61.00 kcal/mol)
- IQR: ≈7 kcal/mol higher than DJM (51.1 kcal/mol)

CA8:

- Median free energy almost 16 kcal/mol more favorable than DJM (−62.7 kcal/mol)
- IQR: ≈15 kcal/mol higher than DJM (59.8 kcal/mol)

CA12:

- Median free energy almost 13.8 kcal/mol more favorable than DJM (−60.4 kcal/mol)
- IQR: ≈10 kcal/mol higher than DJM (54.9 kcal/mol)

**Figure 5:**
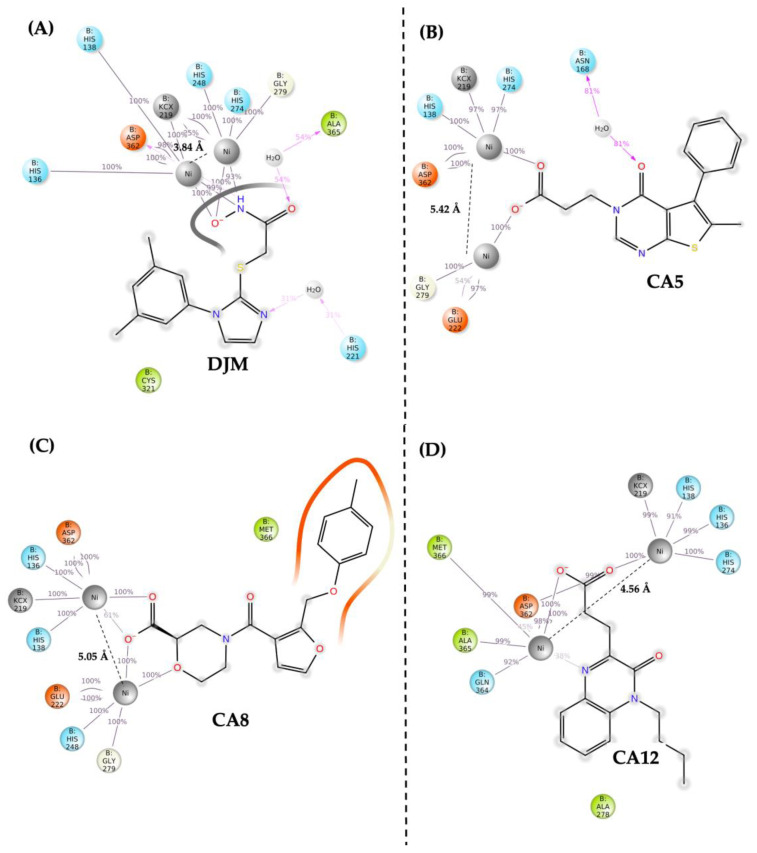
Intermolecular interaction patterns: Chemical structures of the inhibitor DJM (**A**) and candidate inhibitors CA5 (**B**), CA8 (**C**), and CA12 (**D**) are displayed, along with non-bonding interactions involving nickel ions and protein residues within the urease binding site. Residue colors signify the nature of the interaction: orange for negatively charged (ionic), light blue for positively charged (ionic), gray for nickel ions and carbamylated residue Lys219, green for hydrophobic, and light yellow for glycine. The numerical representation of interaction frequency is depicted in light gray, providing quantitative insights into the occurrence of specific interactions. Additionally, the distances of the nickel ions are visually represented in black.

In the final analysis, the networks of intermolecular interactions between the urease enzyme and both the control DJM and the inhibitor candidates CA5, CA8, and CA12 were comprehensively calculated. This evaluation aimed at identifying conserved residues and interactions crucial for the inhibitory activity of these compounds. All three inhibitor candidates separate nickel ions by more than 1 Å compared with DJM, as illustrated in Figure 5. Similar to DJM, the inhibitor candidates feature a structural region with a carboxylic acid moiety. This acid group coordinates with nickel ions and interacts with the basic residues His136, His138, and the acidic residue Asp362. Additionally, other residues, such as His248 and His274, appear to contribute to coordination, although less frequently.

The exploration of interactions between protein–ligand complexes involving metal ions as cofactors constitutes a pivotal aspect of drug design and efficacy. In this context, our work analyzed the interplay between potential pharmaceutical compounds and nickel ions, crucial cofactors known for their involvement with residues in target proteins.

Our results show distinct structural elements in the pharmacophore, encompassing nitrogenous heterocycles, amide groups, and carboxyl groups. However, the majority of interactions between the cofactor and the DJM occur in the hydroxycetamide groups, this group has the capacity to generate coordination bonds with metals ions, as demonstrated previously [24]. The candidates found are rich in oxygen (O) and nitrogen (N), generating possible chelating sites to coordinate metal ions. The coordination of metals in amides and carboxylic acids is facilitated by the electron-rich nature and close proximity of the N and O atoms within these functional groups. The N and O possess readily accessible pairs of electrons that can coordinate with metal ions. The disparity in electronegativity between these atoms further enables coordinate bonds with metal cations [25]. Moreover, the spatial arrangement of atoms within the amide and carboxylic acid functional groups facilitates efficient interaction with metal centers, making them excellent ligands. DJM possesses other atoms, such as sulfur, capable of coordinating with metals [26] and nitrogenous heterocycles. However, the presence of bulky heterocyclic groups in close proximity may impede coordination with active sites, likely due to steric hindrance, although aromatic rings, typically contributing to system stability, experience planarity distortion in the presence of significant steric hindrance [27].

For the analysis of our candidates, the results indicate that the intermolecular interaction patterns of the chemical structures CA5, CA8, and CA12, unlike DJM, occur in the amide and carboxyl groups. Effective interaction around the carboxyl group facilitates interaction with nickel ions, particularly in CA5. A comparative analysis was conducted with CA12, demonstrating nickel interaction primarily through the O atoms of carboxyl groups, with minimal involvement of amide functionalities. This distinction emphasizes the diverse mechanisms by which these compounds can interact with nickel ions. For this reason, the interaction with nickel ions is confined to the carboxyl group in CA5 and CA12. On the other hand, in contrast with the other results, CA8 exhibits a unique interaction pattern. In this case, nickel ions interact not only with the carboxyl group but also with an O atom of a heterocycle located two carbon atoms away. This arrangement enables a chelation mechanism akin to polyphenol–metal interactions, showcasing the versatility of our findings.

**Figure 6:**
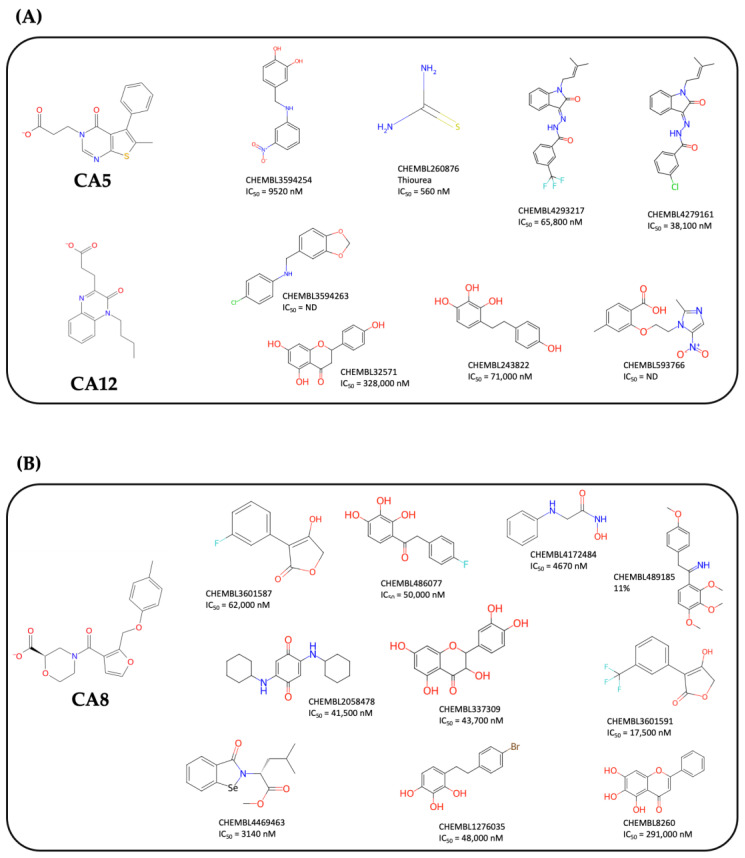
Comparison between urease inhibitors (UIs) for *Hp* deposited in ChEMBL and our candidates. From the multidimensional analysis carried out in PCA, UMAP and t-SNE, two main similarity clusters were detected: (**A**) UIs similar to candidates 5 and 12 and (**B**) UIs similar to candidate 8.

Finally, the multidimensional analysis over the chemical space of *Hp* UIs shows that the chemical nature of candidates 5 and 12 are more similar to each other than to candidate 8. The three methods (PCA, t-SNE and UMAP) used show coincident results, finding molecules from the chemical space of UI that show similarities with the CA5 and CA12 groups on the one hand and CA8 on the other. While PCA provides a linear projection of the components generated by reducing the dimensionality of the analyzed data, t-SNE and UMAP are capable of non-linearly identifying similarity relationships. Even when using a semi-supervised method (tailored), where we label whether the compounds belong to UIs (gray dots in Figure S6) or are our candidates (red dots in Figure S6), the methods are able to find similarities in both of the abovementioned groups. Specifically, candidates 5 and 12 show high physicochemical similarities with eight compounds. Figure 6A shows the ChEMBL code and the inhibition value (IC_50_) of said compounds experimentally tested in urease enzyme. Among these, thiourea [28], a classic UI that is highly potent but toxic in humans, stands out. Additionally, oxoindolines [29], diarylethane [30], isoflavones [31] and flavonoids [32] can be found. For the cluster made up of compound 8 (Figure 6B), we can find 10 compounds of similar nature according to our analyses. In this group we find arylfurans [33], hydroaxamic acids [34], benzoquinones [35], flavonoids [32], cathecols [36], benzisoselenazol [37], and deoxybenzoins [38]. Within the latter, the compounds that present the greatest inhibition in *Hp*U are hydroxamic acid with 4670 nM and a benzisoselenazole with 3140 nM.

## 3. Materials and Methods

### 3.1. Molecules Database Refinement

For the refinement of the molecules database, a set of small molecules was retrieved from the ZINC15 database and subjected to several preliminary filters, such as molecules unique available to purchase, LogP ≤ 5, and MW ≤ 500 g/mol. Subsequently the database was manually cured by using LigPrep. Afterwards, the “Rule of Five” (Lipinski) to assess basic pharmacokinetic properties was used [39]. The subsequent step involved evaluating the absorption, distribution, metabolism, and excretion (ADME) properties using the “Rule of Three” [40]. Furthermore, the bioavailability assessment was conducted using the “Rule of Veber,” which considers parameters relevant to oral drug absorption [41]. Finally, the database was analyzed with Qikprop tools which allowed the obtention of 39 additional features.

### 3.2. Pharmacophore-Based Virtual Screening

An extensive search was conducted on the Web of Science (WOS) database, using the keywords “urease inhibitors” AND “*Helicobacter pylori*” AND “coumarins”, to explore scientific literature. The search was restricted to articles published from 2010 onwards, also considering seminal articles. The compounds that lacked inhibition measurement through half-maximal inhibitory concentration (IC_50_) calculation were excluded. Subsequently, these molecules were prepared with LigPrep in the same conditions as those of the ZINC15 database. These reported UIs were employed in order to build ten pharmacophore hypotheses with the phase tool [18], considering HB donors and HB acceptors, charged groups, hydrophobic groups, and aromatic rings. According to the BEDROC metric, the best two pharmacophore hypotheses were applied to the initial database. After the calculation of the PSS term, only 16% of compounds showed an improved value and were used for further analysis.

### 3.3. Protein Receptor Preparation

The three-dimensional (3D) structure of the urease enzyme from *Hp* (PDB ID: 6ZJA) was selected after conducting a comprehensive analysis of the RCSB database. A single monomer comprising α and β subunits was extracted from the urease enzyme’s dodecamer assembly to ensure functional activity. The preservation of the active site, including the nickel ions, was a primary consideration. The protein preparation process was carried out using the Maestro–Schrödinger suite. Hydrogens were added, and the protonation state was assigned at pH 7.2 using Propka v.3.1 [42]. Subsequently, a brief energy minimization was performed to reduce any major steric clashes between atoms.

### 3.4. Structure-Based Virtual Screening and MM-GBSA Calculations

Firstly, we identified whether the glide program can redock the inhibitors into their respective three-dimensional urease structures with high similarity to the crystallographic pose (PDB ID: 6ZJA for DJM, 6QSU for BME and 1E9Y for HAE). Then, we proceeded to repeat the process but in the 6ZJA entry.

To conduct VS, the receptor utilized was the urease enzyme, including the previously prepared nickel ions. The protein was duplicated to build two variants, one with the Lys219 carbamide (denominated KCX) and a second receptor with a neutral Lys219 (denominated LYN). The screening grid consisted of two parts, a smaller, centered grid (15 Å × 15 Å × 15 Å) and a larger grid (36 Å × 36 Å × 36 Å). The center of the grid was determined by a centroid created from the coordinates of the key *Hp*U residues that form the catalytic site (H136, H138, KCX219/LYN219, H221, H248, H274, C322, R338, D362) and the nickel ions. The best candidates selected from the PBVS were used as input to perform three rounds of VS by using the following scoring functions of glide consecutively: HTVS, SP, and XP (see the complete protocol in Figure 1) with OPLS3e as force field [43]. In each round, the best fraction of candidates was selected according to the docking energies. Subsequently, and with the purpose of identifying inhibitor candidates with chelating potential, those molecules that were more than 3.5 angstrom (Å) away from any of the nickel ions were discarded.

Finally, the compounds with the best docking scores were selected for MM-GBSA calculations by using the prime [44] tool from the Maestro–Schrödinger suite. The solvent was treated by variable dielectric surface generalized born to adjust the polarizable effect of charges distribution [45]. The molecules were categorized by receptor binding, as follows: KCX, LYN, or Both (those molecules found in KCX and LYN receptors at the same time). We docked and performed MM-GBSA calculations of the following classical inhibitors of urease as controls: DJM, HAE, BME and the natural substrate urea. The lowest binding energy derived from MM-GBSA calculations of these controls was used as cutoff to filter the best candidates in this stage.

### 3.5. ADMET Calculations

The remnant candidates were evaluated with six ADME rules: Ghose, Egan, Muegge, BRENK, PAINS and lead-likeness. We also include previous filters Rule of Five, Jorgensen’s Rule of Three and Veber’s rule. Each molecule was ranked according to the number of violations or alerts, allowing the identification of compounds with adequate pharmacokinetic and toxicological properties.

### 3.6. Molecular Dynamics Simulations & MMGBSA Calculations

Finally, and only in the subset of compounds without any ADME violations, we employed MDs and MM-GBSA calculations to investigate the protein–ligand affinity. We placed each complex into an orthorhombic box filled with pre-equilibrated simple point charge (SPC) water molecules and neutralized them with Na^+^ and Cl^−^ ions. The simulations were performed using the NPT ensemble (temperature = 300 K and pressure = 1 atm). The Desmond package from the Maestro–Schrödinger suite was used to conduct the simulations and OPLS3e as force field. The Desmond package performs six stages of minimization and short MDs as a default protocol for equilibration/relaxation, totalizing 60 ps and a Brownian dynamic. Following this, 100 ns of full-atomistic production MDs were conducted. To provide a basis for comparison, we also performed MDs of the urease–DJM, urease–HAE, and urease–BME complexes under the same conditions.

Upon the completion of the MDs, we assessed the thermodynamic stability by calculating the RMSD along the entire trajectory. Simultaneously, we measured the protein–ligand energy interactions at each frame of the simulation using the MM-GBSA method. Our analysis focused solely on candidates with a binding energy lower than the urease–DJM complex and with a stable RMSD profile. These selected candidates were evaluated to determine the intermolecular interactions governing the affinity of the protein–ligand–cofactors complex. Furthermore, these candidates were compared with the chemical space of UIs already discovered through three-dimensional reduction methods. For this, 357 non-redundant *Hp*U inhibitors were downloaded from the ChEMBL database [46] in SMILES format. This dataset was combined with our inhibitor candidates, which was subsequently converted to extended-connectivity fingerprints (ECFPs [47]) using RDKit [48]. ECFPs are 2048-bit vectors that store information about each molecule based on the presence or absence of different physicochemical characteristics. Subsequently, the high-dimensional data were analyzed with three methods, principal component analysis (PCA), t-distributed stochastic neighbor embedding (t-SNE) and uniform manifold approximation and projection (UMAP), from the ChemPlot library in Python [49]. This analysis allows us to compare our candidates with already reported inhibitors, based on a similarity calculated from multiple common characteristics in a semi-supervised (tailored) manner.

## 4. Conclusions

Our comprehensive study aimed at identifying novel UIs for *Hp* involved a multi-methodological approach, combining PBVS, SBVS, MDs, MM-GBSA energy calculations, ADME evaluations and multidimensional analysis over the chemical space of UIs reported.

In the initial phases, we meticulously curated a vast molecular database, applying stringent filters to ensure the selection of compounds with favorable pharmacokinetic properties. PBVS, guided by known UIs, led to identifying potential candidates, further refined through SBVS using HTVS, SP, and XP algorithms. Notably, subsets “Both_KCX” and “LYN_Both” were scrutinized, revealing differential interaction patterns with the LYN and KCX variants of Lys219, a crucial residue in urease. This nuanced understanding of binding preferences informed the selection of promising drug candidates. Energetic re-scoring using MM-GBSA highlighted compounds within subsets “KCX_Both” and “KCX_Only” as particularly promising, surpassing the inhibitory potency of classic UIs DJM in the KCX variant. ADME predictions further filtered these candidates, emphasizing those with optimal pharmacological profiles. MDs provided valuable insights into the stability and behavior of selected compounds, with CA5, CA8, and CA12 demonstrating notable affinity and stability comparable to the control DJM. Finally, the candidates proposed here show physicochemical similarities with inhibitors already reported in the literature, mostly heterocycles, with inhibition ranges of nM. Two of our compounds also have similarities with thiourea, another classic UI that has great ureolytic potency but is toxic in humans, unlike our compounds, which approved more than eight pharmacokinetic/pharmacodynamic rules (with more than 20 criteria embedded in these rules).

In conclusion, our integrative approach identified potential UIs with promising pharmacological characteristics. The nuanced understanding of interaction patterns and the thorough evaluation of compound stability and ADMET profiles contribute valuable insights to the ongoing efforts to develop effective therapies against *Helicobacter pylori* infections. These findings lay a foundation for future experimental validations and drug development endeavors in the field.

## Figures and Tables

**Table 1:** Number of molecules obtained in the SBVS protocol.

Docking Algorithm	Input Molecules	Output Docking Poses	Cutoff	Preselected Molecules	Molecules without the Binding Site	Selected Molecules	Percent
**LYN variant**							
HTVS	234,607	552,716	<−4.5	156,814	13,345	143,469	26
SP	143,469	429,972	<−6.5	93,829	67	93,762	22
XP	93,762	267,641	<−8.5	11,368	0	11,368	4
**KCX variant**							
HTVS	234,607	600,588	<−4.5	196,349	9901	186,448	31
SP	186,448	557,590	<−6.5	105,364	138	105,226	19
XP	105,226	297,494	<−8.5	16,160	0	16,160	3

The percentage column shows the percentage of selected molecules with respect to the input molecules for a specific docking algorithm.

## Data Availability

Data are contained within the article and supplementary material.

## Supplementary Materials

The following supporting information can be downloaded at: https://www.mdpi.com/article/10.3390/ijms25041968/s1.

## References

[1] Kusters J.G., Van Vliet A.H.M., Kuipers E.J. (2006). Pathogenesis of *Helicobacter pylori* infection. Clin. Microbiol. Rev..

[2] Baj J., Forma A., Sitarz M., Portincasa P., Garruti G., Krasowska D., Maciejewski R. (2021). *Helicobacter pylori* virulence factors—Mechanisms of bacterial pathogenicity in the gastric microenvironment. Cells.

[3] Molaoa S.Z. (2021). Prevalence *of Helicobacter pylori* infection and the incidence of the associated malignant and peptic ulcer disease (PUD) at Nelson Mandela Academic Hospital: A retrospective analysis. J. Drug Assess..

[4] Smith S., Fowora M., Pellicano R. (2019). Infections with *Helicobacter pylori* and challenges encountered in Africa. World J. Gastroenterol..

[5] Uotani T., Miftahussurur M., Yamaoka Y. (2015). Effect of bacterial and host factors on *Helicobacter pylori* eradication therapy. Expert. Opin. Ther. Targets.

[6] Vaira D., Fiorini G., Zullo A., Gatta L., Castelli V., Ricci C., Cassol F. (2012). Newer agents for *Helicobacter pylori* eradication. Clin. Exp. Gastroenterol..

[7] Lage T.C.A., Maciel T.M.S., Mota Y.C.C., Sisto F., Sabino J.R., Santos J.C.C., Figueiredo I.M., Masia C., de Fátima A., Fernandes S.A. (2018). In vitro inhibition of *Helicobacter pylori* and interaction studies of lichen natural products with jack bean urease. New J. Chem..

[8] Rego Y.F., Queiroz M.P., Brito T.O., Carvalho P.G., de Queiroz V.T., de Fatima A., Macedo F. (2018). A review on the development of urease inhibitors as antimicrobial agents against pathogenic bacteria. J. Adv. Res..

[9] Zhao H., Wu Y., Xu Z., Ma R., Ding Y., Bai X., Rong Q., Zhang Y., Li B., Ji X. (2019). Mechanistic insight into the interaction between *helicobacter pylori* urease subunit a and its molecular chaperone Hsp60. Front. Microbiol..

[10] Olofsson A., Vallström A., Petzold K., Tegtmeyer N., Schleucher J., Carlsson S., Haas R., Backert S., Wai S.N., Gröbner G. (2010). Biochemical and functional characterization of *Helicobacter pylori* vesicles. Mol. Microbiol..

[11] Tao R., Li J., Guan Y., Liang Y., Hu B., Lv J., Chu G. (2018). Effects of urease and nitrification inhibitors on the soil mineral nitrogen dynamics and nitrous oxide (N2O) emissions on calcareous soil. Environ. Sci. Pollut. Res..

[12] Martins M., Sant’anna S., Zaman M., Santos R., Monteiro R., Alves B., Jantalia C., Boddey R., Urquiaga S. (2017). Strategies for the use of urease and nitrification inhibitors with urea: Impact on N_2_O and NH_3_ emissions, fertilizer-15N recovery and maize yield in a tropical soil. Agric. Ecosyst. Environ..

[13] Hughes J.P., Rees S.S., Kalindjian S.B., Philpott K.L. (2011). Principles of early drug discovery. Br. J. Pharmacol..

[14] Kafarski P., Talma M. (2018). Recent advances in design of new urease inhibitors: A review. J. Adv. Res..

[15] Asghar H., Asghar H., Asghar T. (2021). A Review on Anti-urease Potential of Coumarins. Curr. Drug Targets.

[16] Sterling T., Irwin J.J. (2015). ZINC 15*—*Ligand Discovery for Everyone. J. Chem. Inf. Model..

[17] Maestro S. (2020–2021). Schrödinger Release 2021-1.

[18] Dixon S.L., Smondyrev A.M., Knoll E.H., Rao S.N., Shaw D.E., Friesner R.A. (2006). PHASE: A new engine for pharmacophore perception, 3D QSAR model development, and 3D database screening: 1. Methodology and preliminary results. J. Comput. Aided Mol. Des..

[19] Halgren T.A., Murphy R.B., Friesner R.A., Beard H.S., Frye L.L., Pollard W.T., Banks J.L. (2004). Glide: A New Approach for Rapid, Accurate Docking and Scoring. 2. Enrichment Factors in Database Screening. J. Med. Chem..

[20] Friesner R.A., Murphy R.B., Repasky M.P., Frye L.L., Greenwood J.R., Halgren T.A., Sanschagrin P.C., Mainz D.T. (2006). Extra precision glide: Docking and scoring incorporating a model of hydrophobic enclosure for protein-ligand complexes. J. Med. Chem..

[21] Cunha E.S., Chen X., Sanz-Gaitero M., Mills D.J., Luecke H. (2021). Cryo-EM structure of *Helicobacter pylori* urease with an inhibitor in the active site at 2.0 Å resolution. Nat. Commun..

[22] Ha N.C., Oh S.T., Sung J.Y., Cha K.A., Lee M.H., Oh B.H. (2001). Supramolecular assembly and acid resistance of *Helicobacter pylori* urease. Nat. Struct. Biol..

[23] Zhou J.-T., Li C.-L., Tan L.-H., Xu Y.-F., Liu Y.-H., Mo Z.-Z., Dou Y.-X., Su R., Su Z.-R., Huang P. (2017). Inhibition of *Helicobacter pylori* and its associated urease by Palmatine: Investigation on the potential mechanism. PLoS ONE.

[24] Leporati E. (1986). Complex formation equilibria between 2-amino-N-hydroxyacetamide and 2–amino-N-hydroxypentanamide and cobalt (II), nickel(II), copper(II), and hydrogen ions in aqueous solutions. J. Chem. Soc. Dalton Trans..

[25] Moltved K.A., Kepp K.P. (2019). The chemical bond between transition metals and Oxygen: Electronegativity, d-Orbital Effects, and Oxophilicity as Descriptors of Metal-Oxygen Interactions. J. Phys. Chem. C.

[26] Khan S.A., Shahid S., Kanwal S., Hussain G. (2018). Synthesis characterization and antibacterial activity of Cr (III), Co (III), Fe (II), Cu (II), Ni (III) complexes of 4-(2-(((2-hydroxy-5-nitrophenyl) diazenyl) (phenyl) methylene) hydrazinyl) benzene sulfonic acid based formazan dyes and their applications on leather. Dye Pigment..

[27] Verma C., Alfantazi A., Quraishi M.A., Rhee K.Y. (2023). Significance of Hammett and Taft substituent constants on bonding potential of organic corrosion inhibitors: Tailoring of reactivity and performance. Coord. Chem. Rev..

[28] Menteşe E., Bektaş H., Sokmen B.B., Emirik M., Çakır D., Kahveci B. (2017). Synthesis and molecular docking study of some 5,6-dichloro-2-cyclopropyl-1H-benzimidazole derivatives bearing triazole, oxadiazole, and imine functionalities as potent inhibitors of urease. Bioorg. Med. Chem. Lett..

[29] Yang Y.-S., Su M.-M., Zhang X.-P., Liu Q.-X., He Z.-X., Xu C., Zhu H.-L. (2018). Developing potential *Helicobacter pylori* urease inhibitors from novel oxoindoline derivatives: Synthesis, biological evaluation and in silico study. Bioorg. Med. Chem. Lett..

[30] Xiao Z.-P., Shi W.-K., Wang P.-F., Wei W., Zeng X.-T., Zhang J.-R., Zhu N., Peng M., Peng B., Lin X.-Y. (2015). Synthesis and evaluation of N-analogs of 1,2-diarylethane as *Helicobacter pylori* urease inhibitors. Bioorg. Med. Chem..

[31] Xiao Z.P., Shi D.H., Li H.Q., Zhang L.N., Xu C., Zhu H.L. (2007). Polyphenols based on isoflavones as inhibitors of *Helicobacter pylori* urease. Bioorg. Med. Chem..

[32] Xiao Z.-P., Peng Z.-Y., Dong J.-J., He J., Ouyang H., Feng Y.-T., Lu C.-L., Lin W.-Q., Wang J.-X., Xiang Y.-P. (2013). Synthesis, structure-activity relationship analysis and kinetics study of reductive derivatives of flavonoids as *Helicobacter pylori* urease inhibitors. Eur. J. Med. Chem..

[33] Wang X.-D., Wei W., Wang P.-F., Yi L.-C., Shi W.-K., Xie Y.-X., Wu L.-Z., Tang N., Zhu L.-S., Peng J. (2015). Synthesis, molecular docking and biological evaluation of 3-arylfuran-2(5H)-ones as anti-gastric ulcer agent. Bioorg. Med. Chem..

[34] Liu Q., Shi W.-K., Ren S.-Z., Ni W.-W., Li W.-Y., Chen H.-M., Liu P., Yuan J., He X.-S., Liu J.-J. (2018). Arylamino containing hydroxamic acids as potent urease inhibitors for the treatment of *Helicobacter pylori* infection. Eur. J. Med. Chem..

[35] You Z.L., Xian D.M., Zhang M., Cheng X.S., Li X.F. (2012). Synthesis, biological evaluation, and molecular docking studies of 2,5-substituted-1,4-benzoquinone as novel urease inhibitors. Bioorg. Med. Chem..

[36] Xiao Z.P., Ma T.W., Fu W.C., Peng X.C., Zhang A.H., Zhu H.L. (2010). The synthesis, structure and activity evaluation of pyrogallol and catechol derivatives as *Helicobacter pylori* urease inhibitors. Eur. J. Med. Chem..

[37] Macegoniuk K., Grela E., Palus J., Rudzinska-Szostak E., Grabowiecka A., Biernat M., Berlicki Ł. (2016). 1,2-Benzisoselenazol-3(2H)-one Derivatives as a New Class of Bacterial Urease Inhibitors. J. Med. Chem..

[38] Li H.Q., Xiao Z.P., Yin-Luo, Yan T., Lv P.C., Zhu H.L. (2009). Amines and oximes derived from deoxybenzoins as *Helicobacter pylori* urease inhibitors. Eur. J. Med. Chem..

[39] Lipinski C.A., Lombardo F., Dominy B.W., Feeney P.J. (2012). Experimental and computational approaches to estimate solubility and permeability in drug discovery and development settings. Adv. Drug Deliv. Rev..

[40] Ntie-Kang F., Lifongo L.L., Judson P.N., Sippl W., Efange S.M.N. (2014). How ‘drug-like’ are naturally occurring anti-cancer compounds?. J. Mol. Model..

[41] Veber D.F., Johnson S.R., Cheng H.Y., Smith B.R., Ward K.W., Kopple K.D. (2002). Molecular properties that influence the oral bioavailability of drug candidates. J. Med. Chem..

[42] Olsson M.H.M., SØndergaard C.R., Rostkowski M., Jensen J.H. (2011). PROPKA3: Consistent treatment of internal and surface residues in empirical pKa predictions. J. Chem. Theory Comput..

[43] Roos K., Wu C., Damm W., Reboul M., Stevenson J.M., Lu C., Dahlgren M.K., Mondal S., Chen W., Wang L. (2019). OPLS3e: Extending Force Field Coverage for Drug-Like Small Molecules. J. Chem. Theory Comput..

[44] Jacobson M.P., Pincus D.L., Rapp C.S., Day T.J.F., Honig B., Shaw D.E., Friesner R.A. (2004). A Hierarchical Approach to All-Atom Protein Loop Prediction. Proteins Struct. Funct. Genet..

[45] Li J., Abel R., Zhu K., Cao Y., Zhao S., Friesner R.A. (2011). The VSGB 2.0 Model: A Next Generation Energy Model for High Resolution Protein Structure Modeling. Proteins.

[46] Mendez D., Gaulton A., Bento A.P., Chambers J., de Veij M., Félix E., Magariños M.P., Mosquera J.F., Mutowo P., Nowotka M. (2019). ChEMBL: Towards direct deposition of bioassay data. Nucleic Acids Res..

[47] Rogers D., Hahn M. (2010). Extended-connectivity fingerprints. J. Chem. Inf. Model..

[48] RDKit: Open-Source Cheminformatics. https://www.rdkit.org.

[49] Sorkun M.C., Mullaj D., Koelman J.M.V.A., Er S. (2022). ChemPlot, a Python Library for Chemical Space Visualization**. Chemistry-Methods.

