# Supplementary material for: Unveiling Novel Urease Inhibitors for Helicobacter pylori: A Multi-Methodological Approach from Virtual Screening and ADME to Molecular Dynamics Simulations
Source: Int J Mol Sci. 2024 Feb 6;25(4):1968. doi: 10.3390/ijms25041968 (PMC10888695; doi:10.3390/ijms25041968)
Supplement: Supplementary file 1 [file ijms-25-01968-s001.zip › ijms-2802583-supplementary.pdf]

Article

Unveiling Novel Urease Inhibitors for *Helicobacter pylori*: A Multi-Methodological Approach from Virtual Screening and ADME to Molecular Dynamics Simulations

Paulina Valenzuela-Hormazabal [1,†], Romina V. Sepúlveda [2,†], Melissa Alegría-Arcos [3], Elizabeth Valdés-Muñoz [4], Víctor Rojas-Pérez [4], Ileana González-Bonet [5], Reynier Suardíaz [6], Christian Galarza [7], Natalia Morales [8], Verónica Leddermann [8], Ricardo I. Castro [9], Bruna Benso [10], Gabriela Urra [11], Erix W. Hernández-Rodríguez [11,12,*] and Daniel Bustos [11,*]

[1] Departamento de Farmacología, Facultad de Ciencias Biológicas, Universidad de Concepción, Concepción 4030000, Chile; paulinvalenzuela@udec.cl
[2] Center for Bioinformatics and Integrative Biology, Facultad de Ciencias de la Vida, Universidad Andres Bello, Av. República 330, Santiago 8370146, Chile; romina.sepulveda@unab.cl
[3] Núcleo de Investigación en Data Science, Facultad de Ingeniería y Negocios, Universidad de las Américas, Santiago 7500000, Chile; malegriaa@udla.cl
[4] Doctorado en Biotecnología Traslacional, Facultad de Ciencias Agrarias y Forestales, Universidad Católica del Maule, Talca 3480094, Chile; elizabeth.valdes@alu.ucm.cl (E.V.-M.); victor.rojas.03@alu.ucm.cl (V.R.-P.)
[5] Biomedical Research Labs, Facultad de Medicina, Universidad Católica del Maule, Talca 3480094, Chile; ileanag@ucm.cl
[6] Departamento de Química Física, Facultad de Ciencias Químicas, Universidad Complutense de Madrid, 28040 Madrid, Spain; reysuard@ucm.es
[7] Departamento de Matemáticas, Facultad de Ciencias Naturales y Matemáticas, Escuela Superior Politécnica del Litoral, Guayaquil 090112, Ecuador; chedgala@espol.edu.ec
[8] Magíster en Ciencias de la Computación, Universidad Católica del Maule, Talca 3460000, Chile; nmoralesr@ucm.cl (N.M.); vlleddermann@ucm.cl (V.L.)
[9] Multidisciplinary Agroindustry Research Laboratory, Instituto de Ciencias Aplicadas, Facultad de Arquitectura, Construcción y Medio Ambiente, Universidad Autónoma de Chile, Cinco Pte. N°1670, Talca 3467987, Chile; ricardo.castro@uautonoma.cl
[10] School of Dentistry, Faculty of Medicine, Pontificia Universidad Católica de Chile, Santiago 7810000, Chile; bruna.benso@uc.cl
[11] Laboratorio de Bioinformática y Química Computacional, Departamento de Medicina Traslacional, Facultad de Medicina, Universidad Católica del Maule, Talca 3480094, Chile; gabriela.urra@alu.ucm.cl
[12] Unidad de Bioinformática Clínica, Centro Oncológico, Facultad de Medicina, Universidad Católica del Maule, Talca 3480094, Chile
* Correspondence: ehernandez@ucm.cl (E.W.H.-R.); dbustos@ucm.cl (D.B.)
† These authors contributed equally to this work.

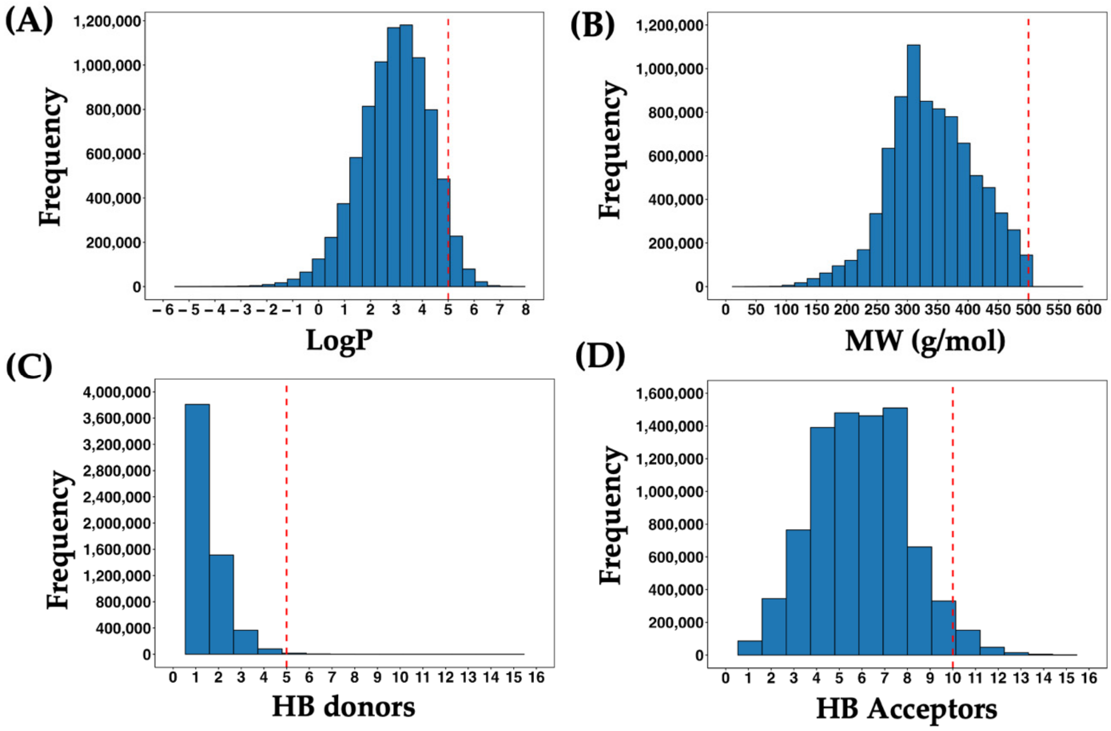

Figure S1. Lipinski's Rule of Five: Each subgraph shows the distribution of values for each parameter of Lipinski's rule: (A) Partition coefficient; (B) Molecular Weight; (C) Hydrogen Bond donors; and (D) Hydrogen Bond acceptors. The dotted red line represents the cutoff value used for each parameter. 5 in (A); 500 g/mol in (B); 5 donors in (C); and 10 acceptors in (D).

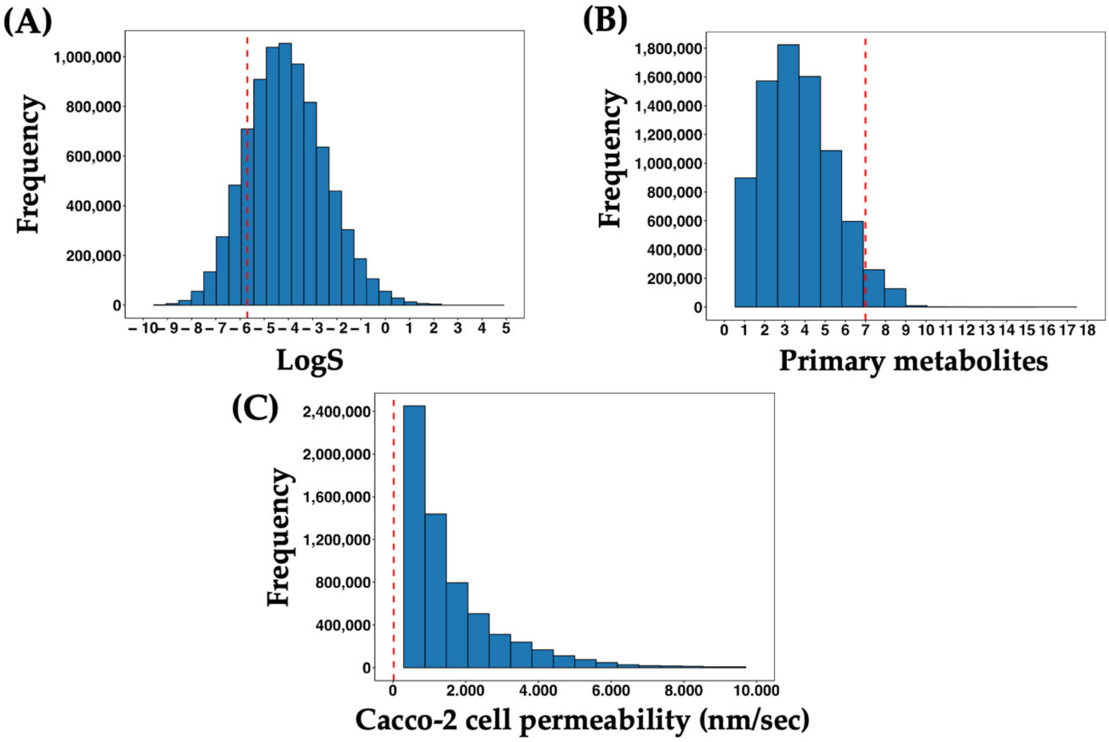

Figure S2. Jorgensen’s Rule of Three: Each subgraph shows the distribution of values for each parameter of Jorgensen's rule: Aqueous solubility in (A); Primary metabolites in (B); and Permeability in Caco-2 cells in (C). The dotted red line represents the cutoff value used for each parameter: -5.7 in (A); 7 metabolites in (B); and 22 nm/sec in (C).

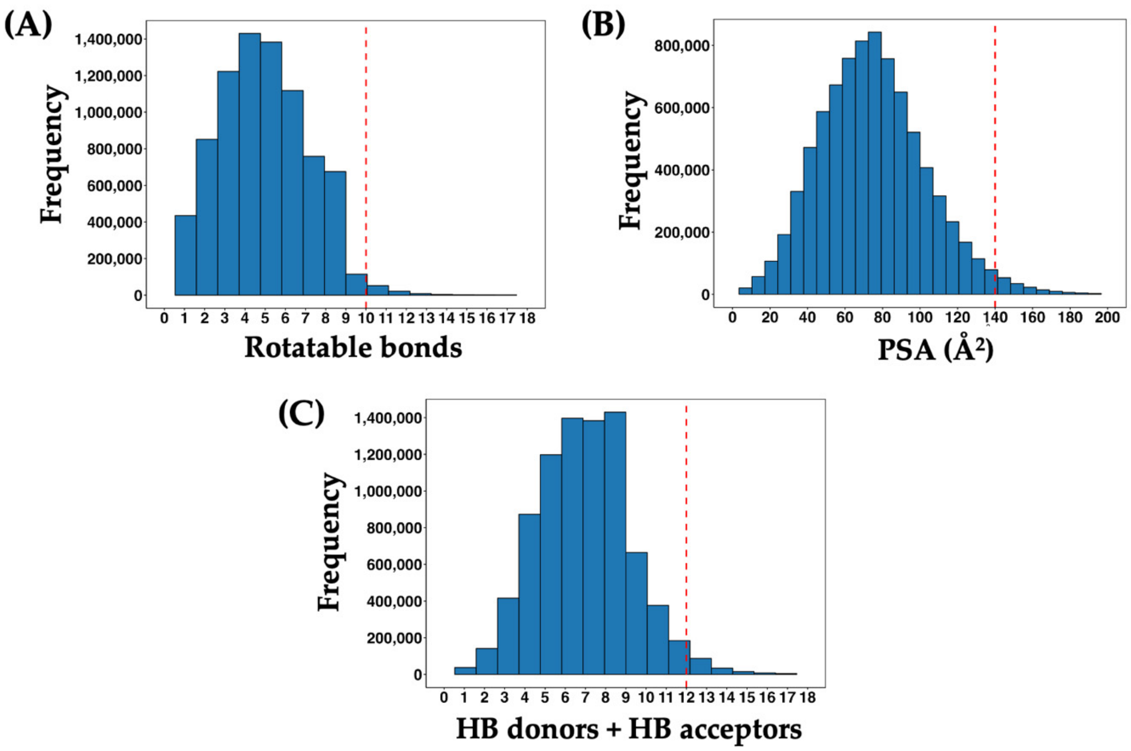

Figure S3. Veber's Rule: Each subgraph shows the distribution of values for each parameter of Veber's rule: Rotatable bonds in (A); Polar Surface Area in (B); and the sum of hydrogen bonds donors and acceptors in (C). The dotted red line represents the cutoff value used for each parameter: 10 rotatable bonds in (A); 140 Å^2 in (B); and 12 donors+acceptors in (C).

Table S1. Redocking process of classical *Hp*U inhibitors used as control.

Chemical controls	Pose number	Docking score	RMSD
BME	1	-3.08	2.72
	2	-2.94	2.32
	3	-2.92	2.40
	4	-2.42	2.74
	5	-2.25	2.58
	6	-2.20	2.31
	7	-2.10	2.41
DJM	1	-9.37	4.56
	2	-8.62	2.46
	3	-8.52	2.17
	4	-8.41	4.22

	5	-8.39	3.98
	6	-8.38	1.80
	7	-8.37	4.17
	8	-8.36	1.82
	9	-8.17	1.81
	10	-8.13	1.86
HAE	1	-2.94	1.93
	2	-2.86	1.63
	3	-2.82	3.01
	4	-2.37	1.97
	5	-2.23	2.48
	6	-2.17	2.19
	7	-2.00	2.93
	8	-1.66	2.60
	9	-1.65	2.53
	10	-1.42	2.84

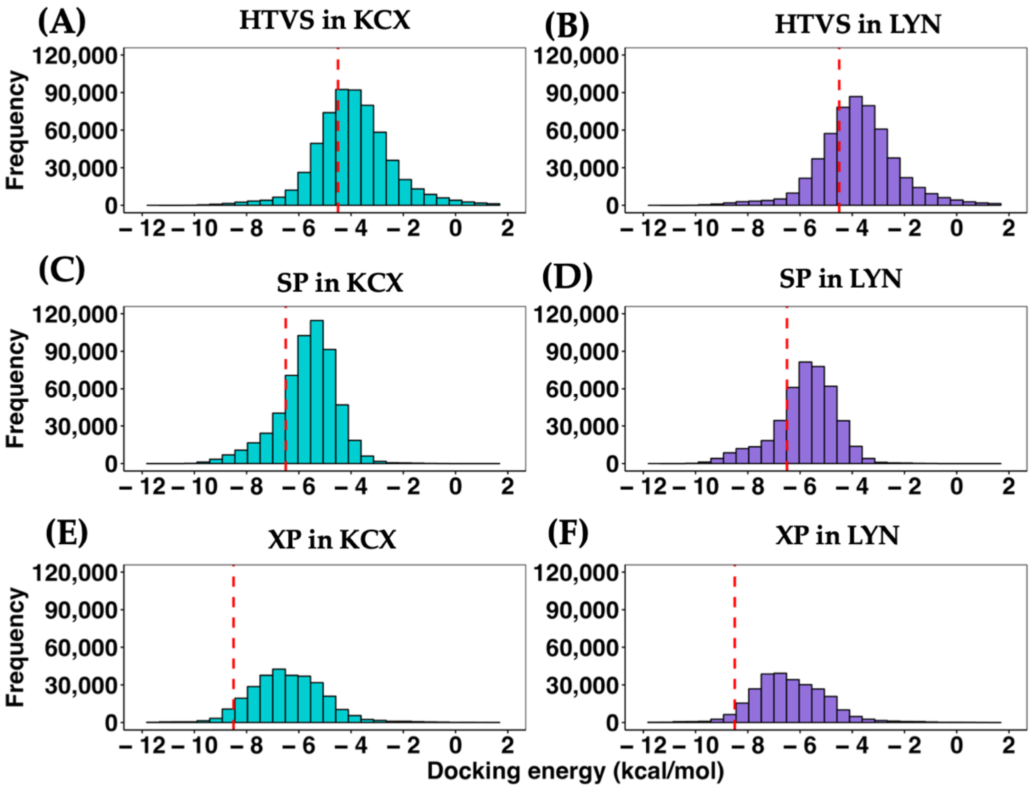

Figure S4. Docking energy distribution at each step of the docking layer: In panels (A) and (B), the docking energies for KCX and LYN are depicted, respectively, computed using the High Throughput Virtual Screening (HTVS) algorithm. Moving on to panels (C) and (D), the figures display the docking energies for KCX and LYN utilizing the Standard Precision (SP) algorithm. Finally, panels (E) and (F) represent the docking energies for KCX and LYN, respectively, employing the Extra Precision (XP) algorithm.

Table S2. Binding affinity calculated with docking and MM-GBSA technique for the classical urease inhibitors and urea substrate.

Chemical controls	IC_{50} (μM)	$\Delta G_{Experimental}$	$\Delta G_{Theorical}$ LYN variant	$\Delta G_{Theorical}$ KCX variant
DJM	19.6	-6.461 kcal/mol	-60.4 kcal/mol	-64.3 kcal/mol
HAE	70.0	-5.702 kcal/mol	-15.6 kcal/mol	-17.4 kcal/mol
BME	13500.0	-2.544 kcal/mol	-11.5 kcal/mol	-10.8 kcal/mol
Urea	-	-	-17.6 kcal/mol	-39.4 kcal/mol

Table S3. Descriptive statistics of binding free energy obtained with docking and MM-GBSA calculations for the four subsets of candidates.

Statistics	KCX_Both	KCX_Only	LYN_Both	LYN_Only
**Q1**	-62.50	-59.50	-13.50	-17.90
**Median**	-43.90	-37.30	-2.50	2.84
**Q3**	-19.60	-18.40	-11.7	15.3
**Average**	-39.5	-38.20	-0.89	1.95
**Standard Deviation**	28.80	27.10	20.9	27.3
**Count of complexes with a free energy ≥ 0 kcal/mol**	15	11	113	98
**Count of complexes with a free energy < 0 kcal/mol**	139	181	139	81
**Count of complexes with a free energy < DJM**	38 (using -64.3 kcal/mol as cutoff)	36 (using -64.3 kcal/mol as cutoff)	0 (using -60.4 kcal/mol as cutoff)	0 (using -60.4 kcal/mol as cutoff)

Table S4. ADMET parameters for the 38 compounds of the KCX_Both subset with binding free energy better than DJM inhibitor in urease enzyme.

#	ZINC database code	ROF (4)	ROT (3)	Ghose (4)	Egan (2)	Veber (3)	Muegge (9)	PAINS	BRENK	Leadli- keness (3)	Sum of violations	Code
1	ZINC000008627328	0	0	0	0	0	0	0	0	0	0	CA1
2	ZINC000002522532	0	0	0	0	0	0	0	0	0	0	CA2
3	ZINC000031169481	0	0	0	0	0	0	0	0	0	0	CA3
4	ZINC000091624618	0	0	0	0	0	0	0	0	0	0	CA4
5	ZINC000007994969	0	0	0	0	0	0	0	0	0	0	CA5
6	ZINC000008731862	0	0	0	0	0	0	0	0	0	0	CA6
7	ZINC000002584177	0	0	0	0	0	0	0	0	0	0	CA7
8	ZINC000100437608	0	0	0	0	0	0	0	0	1	1	
9	ZINC000071769481	0	0	0	0	0	0	0	0	1	1	
10	ZINC000002120368	0	0	0	0	0	0	0	0	1	1	
11	ZINC000004858708	0	0	1	0	0	0	0	0	0	1	
12	ZINC000089286421	0	0	1	0	0	0	0	0	0	1	
13	ZINC000002479300	0	0	0	0	0	0	0	0	1	1	
14	ZINC000013545426	0	0	0	0	0	0	0	0	1	1	
15	ZINC000001117084	0	0	0	0	0	0	0	0	1	1	
16	ZINC000004320287	0	0	0	0	0	0	0	0	1	1	
17	ZINC000002479297	0	1	0	0	0	0	0	0	0	1	
18	ZINC000095095717	0	0	0	0	0	0	0	0	1	1	
19	ZINC000004839614	0	0	0	0	0	0	0	0	1	1	
20	ZINC000051325904	0	0	0	0	0	0	0	0	1	1	
21	ZINC000004320258	0	0	0	0	0	0	0	0	1	1	
22	ZINC000012360390	0	0	0	0	0	0	0	0	1	1	
23	ZINC000009668624	0	0	0	0	0	0	0	0	1	1	
24	ZINC000040286706	0	1	0	0	0	0	0	0	0	1	
25	ZINC000089991200	0	0	0	0	0	0	0	1	1	2	
26	ZINC000199798219	0	0	0	0	0	0	0	1	1	2	
27	ZINC000095532132	0	0	0	0	0	0	0	1	1	2	
28	ZINC000033795588	0	0	0	0	0	0	0	1	1	2	

29	ZINC000262738161	0	1	1	0	0	0	0	0	0	2
30	ZINC000222498800	0	0	0	0	0	0	0	0	2	2
31	ZINC000222659948	0	0	0	0	0	0	0	0	2	2
32	ZINC000006760357	0	0	1	0	0	0	0	0	1	2
33	ZINC000019798822	0	1	0	0	0	0	0	0	1	2
34	ZINC000257233422	0	0	0	0	0	0	0	0	3	3
35	ZINC000001038956	0	0	0	1	0	1	0	0	1	3
36	ZINC000005000999	1	0	0	1	1	0	0	0	0	3
37	ZINC000012706865	0	0	0	0	0	0	0	0	3	3
38	ZINC000004386690	1	0	0	1	0	1	0	0	1	4

The "Sum of Violations" column calculates the total number of violations for each compound based on the rules used in the analysis. In the results table, the 7 compound numbers with no violations are highlighted in green. Additionally, a specific code corresponding to the candidate number in the final ranking is provided for these compounds.

Table S5. ADMET parameters for the 36 compounds of the KCX_Alone subset with binding free energy better than DJM inhibitor in urease enzyme.

#	ZINC database code	ROF (4)	ROT (3)	Ghose (4)	Egan (2)	Veber (3)	Muegge (9)	PAINS	BRENK	Leadli- keness (3)	Sum of violations	Code
1	ZINC000072412074	0	0	0	0	0	0	0	0	0	0	CA8
2	ZINC000072427629	0	0	0	0	0	0	0	0	0	0	CA9
3	ZINC000006751398	0	0	0	0	0	0	0	0	0	0	CA10
4	ZINC000215300901	0	0	0	0	0	0	0	0	0	0	CA11
5	ZINC000003316814	0	0	0	0	0	0	0	0	0	0	CA12
6	ZINC000008731861	0	0	0	0	0	0	0	0	0	0	CA13
7	ZINC000008700584	0	0	0	0	0	0	0	0	0	0	CA14
8	ZINC001560403828	0	0	0	0	0	0	0	0	0	0	CA15
9	ZINC000005810608	0	0	0	0	0	0	0	0	1	1	
10	ZINC000015106043	0	0	0	0	0	0	0	0	1	1	
11	ZINC000216429060	0	0	0	0	0	0	0	0	1	1	
12	ZINC000012360391	0	0	0	0	0	0	0	0	1	1	
13	ZINC000003298752	0	0	0	0	0	0	0	0	1	1	
14	ZINC000219014368	0	0	0	0	0	0	0	0	1	1	
15	ZINC000095387364	0	0	0	0	0	0	0	0	1	1	
16	ZINC000011867201	0	0	0	0	0	0	0	0	1	1	

17	ZINC000072423992	0	0	1	0	0	0	0	0	0	1
18	ZINC000072409234	0	0	1	0	0	0	0	0	0	1
19	ZINC000000614859	0	0	0	0	0	0	0	1	0	1
20	ZINC000003863640	0	0	0	0	0	0	0	1	0	1
21	ZINC000080316223	0	0	0	0	0	0	1	0	1	2
22	ZINC000022289134	0	0	0	1	0	0	0	0	1	2
23	ZINC000080316289	0	0	0	0	0	0	1	0	1	2
24	ZINC000032934908	0	0	0	0	0	0	0	1	1	2
25	ZINC000000204307	0	0	0	1	0	0	0	0	1	2
26	ZINC000004602529	0	0	0	0	0	0	0	0	2	2
27	ZINC000089991199	0	0	0	0	0	0	0	1	1	2
28	ZINC000012360392	0	1	0	0	0	0	0	0	1	2
29	ZINC000023115099	0	0	0	0	0	0	0	0	2	2
30	ZINC000004387901	0	0	0	0	0	0	0	2	0	2
31	ZINC000006758619	0	1	1	0	0	0	0	0	1	3
32	ZINC000001418849	0	1	1	0	0	0	0	0	1	3
33	ZINC000005710763	0	0	0	0	0	0	0	1	2	3
34	ZINC000257314628	0	0	0	0	1	0	0	0	3	4
35	ZINC000001155565	0	0	0	1	1	0	0	0	2	4
36	ZINC000017080370	0	0	0	1	0	0	0	0	3	4

The "Sum of Violations" column calculates the total number of violations for each compound based on the rules used in the analysis. In the results table, the 8 compounds with no violations are highlighted in green. Additionally, a specific code corresponding to the candidate number in the final ranking is provided for these compounds.

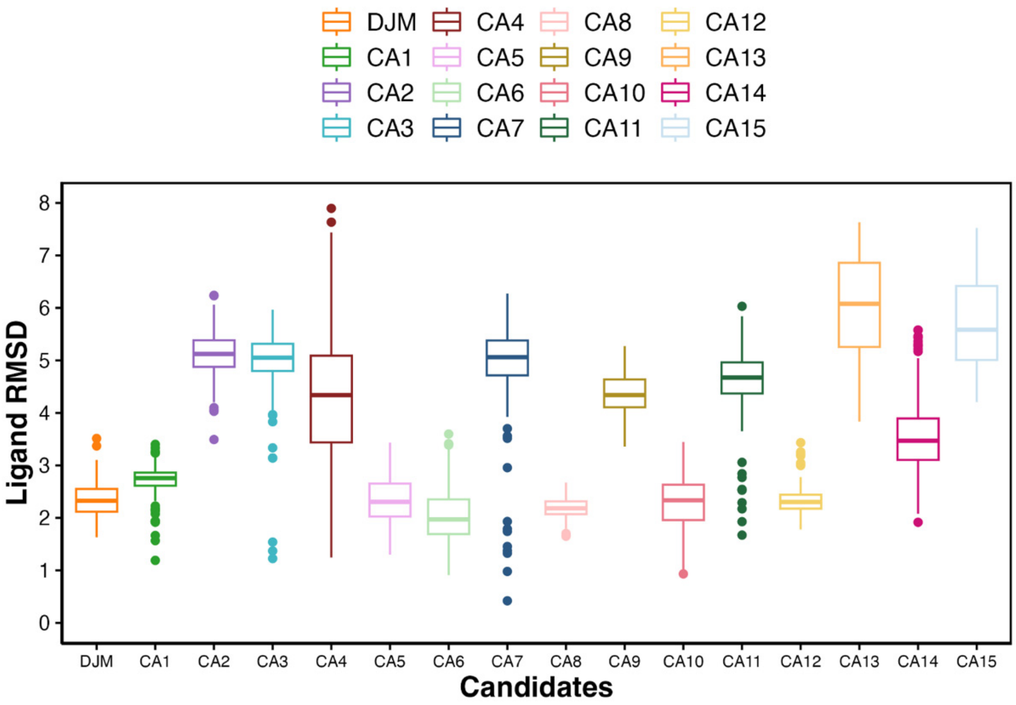

Figure S5. RMSD profiles of urease inhibitor candidates: Each boxplot in the analysis was generated using the RMSD values of the ligand over 200 frames, representing the 100 ns simulation period for each candidate. The control in this comparison is the RMSD of the inhibitor DJM within the urease binding site.

Table S6. Binding affinity calculated with MDs and MM-GBSA technique for the classical urease inhibitors and the VS candidates.

**Compounds**	$\Delta G_{Experimental}$ **(kcal/mol)**	**Median** $\Delta G_{Theorical}$ **(kcal/mol)**	**IQR**$\Delta G_{Theorical}$ **(kcal/mol)**	**Mínimum**$\Delta G_{Theorical}$ **(kcal/mol)**
DJM	-6.461	-46.6	44.2	-117.0
HAE	-5.702	-8.14	4.35	-16.9
BME	-2.544	-0.05	20.9	-39.1
CA1	-		11.0	-53.5
CA2	-		8.12	-53.5
CA3	-		30.9	-103.0
CA4	-		30.1	-87.3
CA5	-		51.1	-142.0
CA6	-		22.2	-73.9
CA7	-		30.3	-90.2
CA8	-		59.8	-156.0
CA9	-		38.8	-126.0

CA10	-	17.0	-75.2
CA11	-	22.2	-68.6
CA12	-	54.9	-147.0
CA13	-	23.1	-97.0
CA14	-	30.7	-92.8
CA15	-	40.1	-145.0

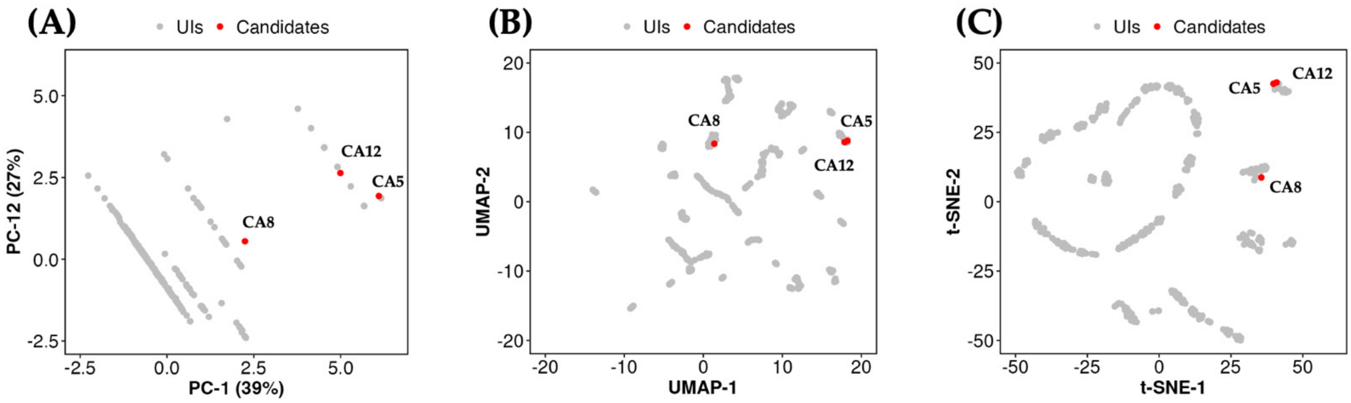

Figure S6. Multidimensional reduction analysis: (A) PCA, (B) UMAP, (C) t-SNE methods were applied into the chemical space comprised by 357 Urease Inhibitors (UIs) depicted in gray dots and the best three candidates (CA5, CA8 and CA11) in red dots. The spatial arrangement and clusters in the plots are calculated from thousands of evaluated physicochemical characteristics.
